# Supplementary figures and images for: Defining cellular diversity at the swine maternal–fetal interface using spatial transcriptomics and organoids
Source: PLoS Biol. 2025 Aug 28;23(8):e3003302. doi: 10.1371/journal.pbio.3003302 (PMC12393714; doi:10.1371/journal.pbio.3003302)

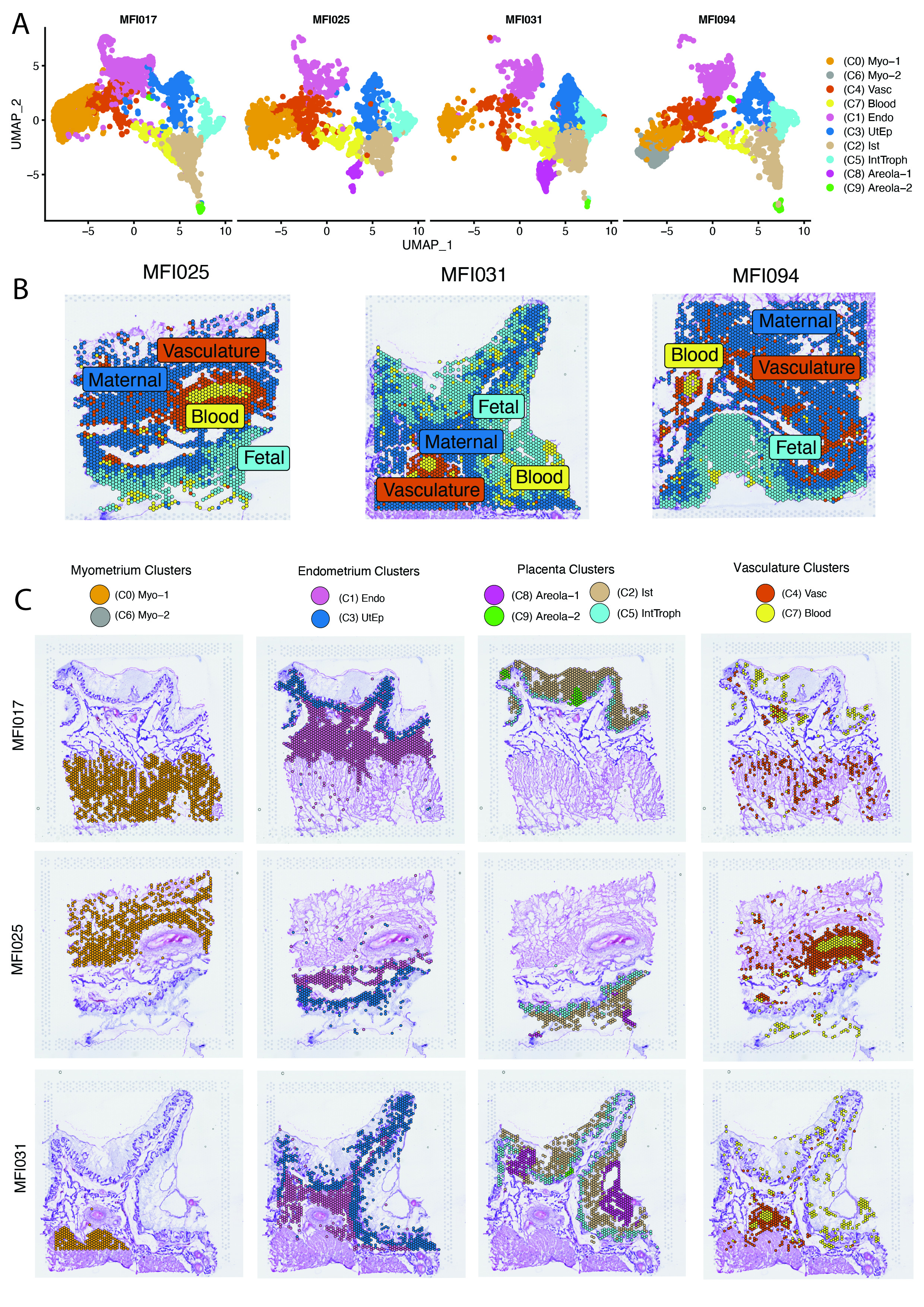

Supplement: S1 Fig — A) UMAP split by sample (MFI). Dots indicate individual visium spots. B) Spatial Dimplot showing separation of maternal and fetal components. Representative maternal–fetal Interface shown. C) Spatial DimPlot separated by sample type and histologic structures showing the localization of UMAP cluster populations. (S1_Fig.JPG) [file pbio.3003302.s001.jpg]

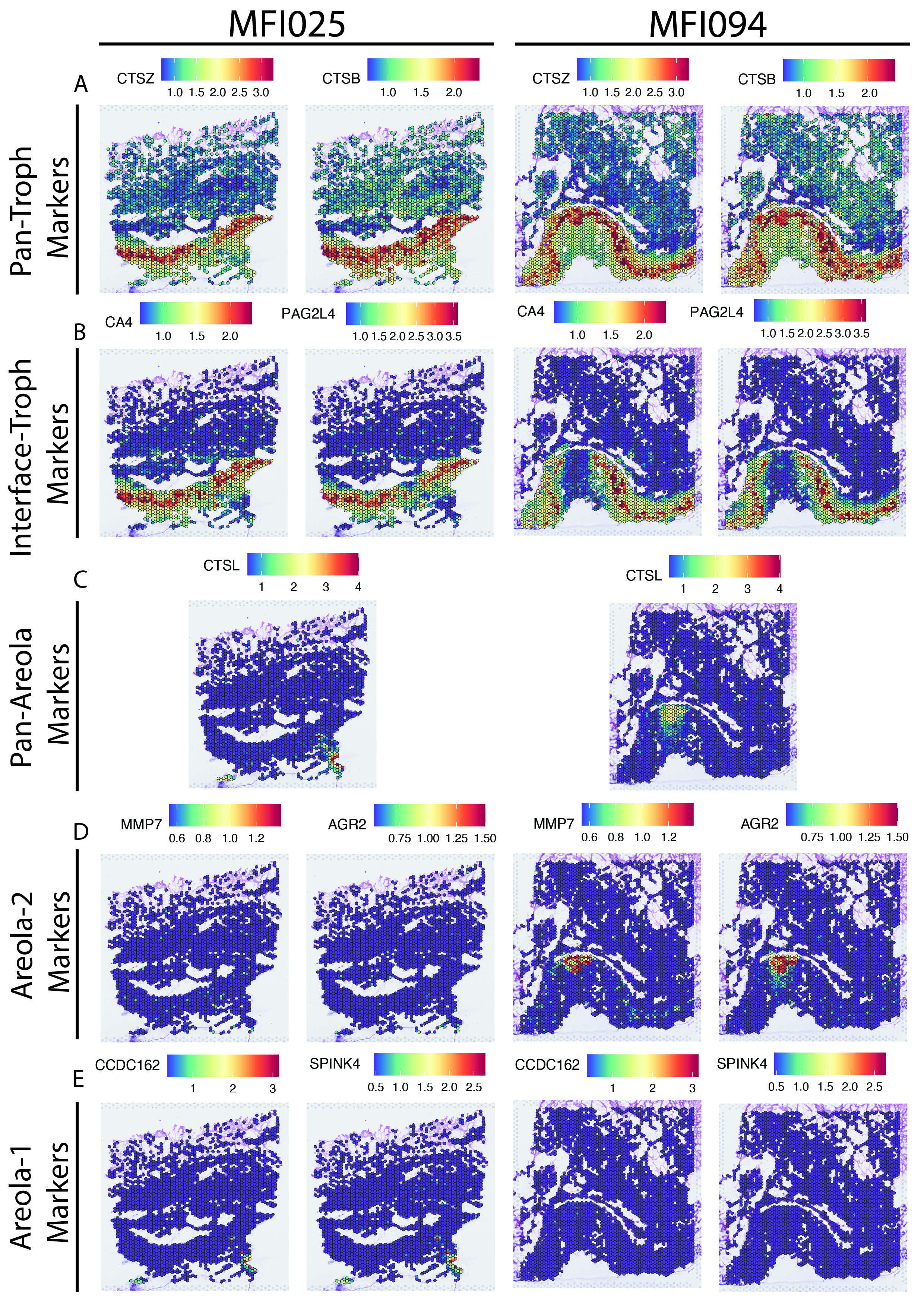

Supplement: S2 Fig — A–E) Spatial feature plots of known and novel markers of various trophoblast populations, split by sample (MFI). Various populations include A) Pan trophoblast markers, B) Interface trophoblast markers, C) Pan-Areola markers, D) Areola-2 markers, and E) Areola-1 markers. (S2_Fig.JPG) [file pbio.3003302.s002.jpg]

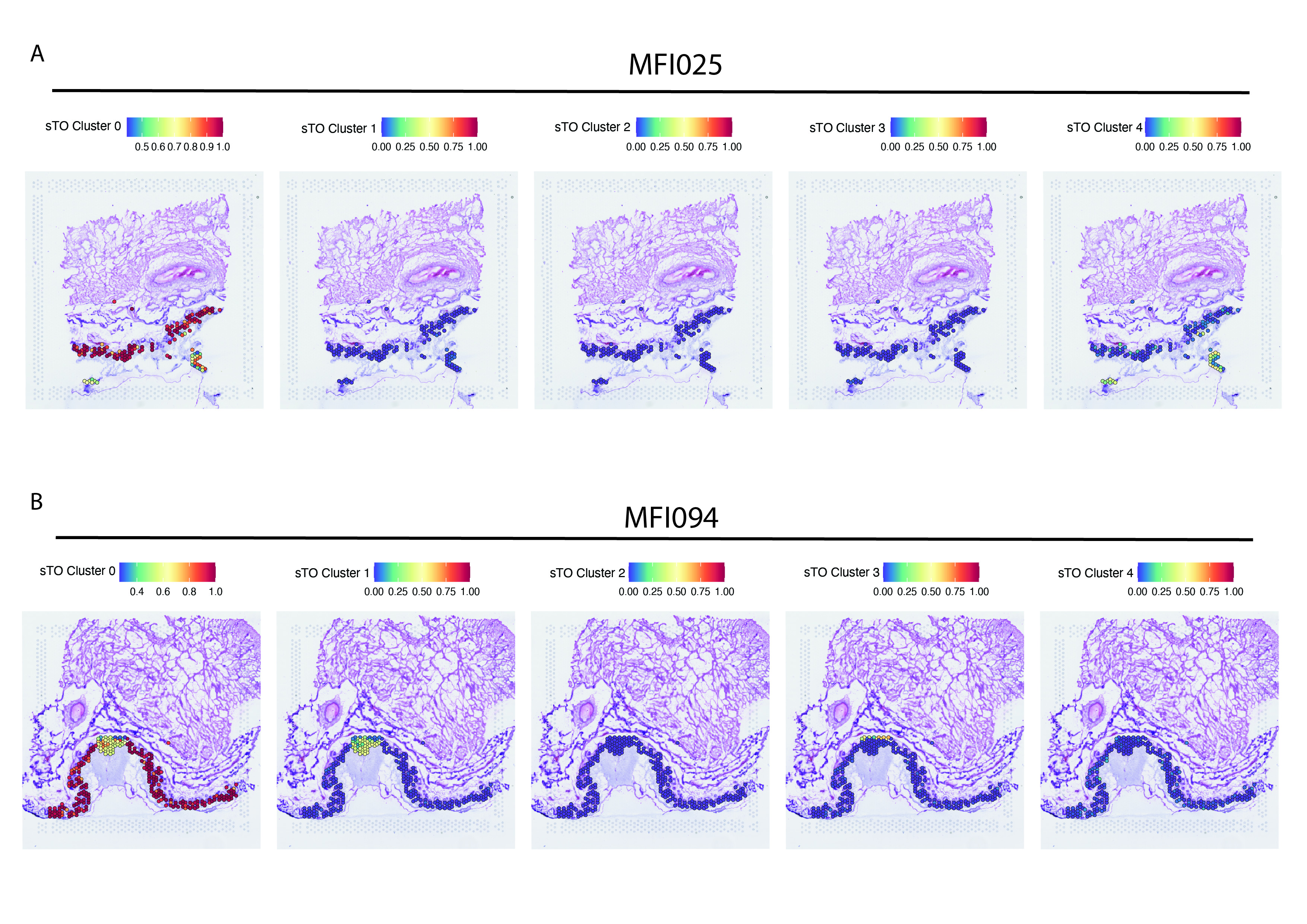

Supplement: S3 Fig — A, B) Spatial feature plot showing prediction scores for each spot in the spatial dataset subsetted by sTO cluster. Color shown indicates probability that the classified sTO cluster is localized to a given position within the spatial dataset. A) Scoring for MFI025. B) Scoring for MFI094. (S3_Fig.JPG) [file pbio.3003302.s003.jpg]

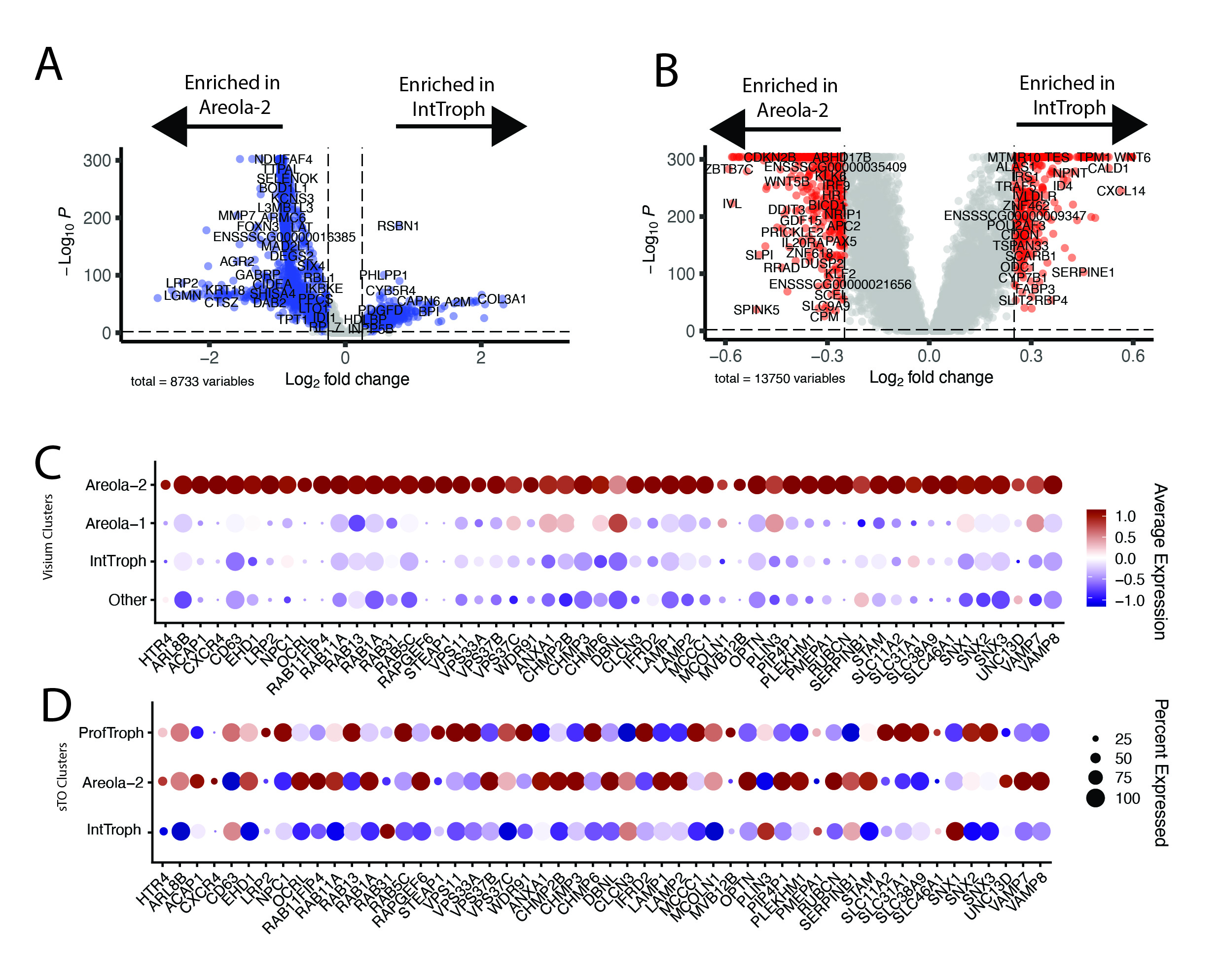

Supplement: S4 Fig — A) Volcano plot showing differentially expressed genes in interface-trophoblasts and areola-2 trophoblasts within the Visium Spatial Transcriptomics data. Significant differences (p < 0.01, Fold-change > 0.25) are shown in blue. B) Volcano plot showing differentially expressed genes in interface-trophoblasts and areola-2 trophoblasts within the sTO single-cell transcriptomics data. Significant differences (p < 0.01, Fold-change > 0.25) are shown in red. C, D) Dotplots showing the expression of endosome-specific genes in both Visium (C) and sTO datasets (D). Plots showing Interface-trophoblast enriched GO-Terms as calculated by DAVID pathway analysis (significant difference = p < 0.05) for Visium spatial transcriptomics (C) and sTO single cell (D). Color of dots denotes average expression, whereas size represents percentage of cells in a cluster expressing the gene of interest. (S4_Fig.JPG) [file pbio.3003302.s004.jpg]

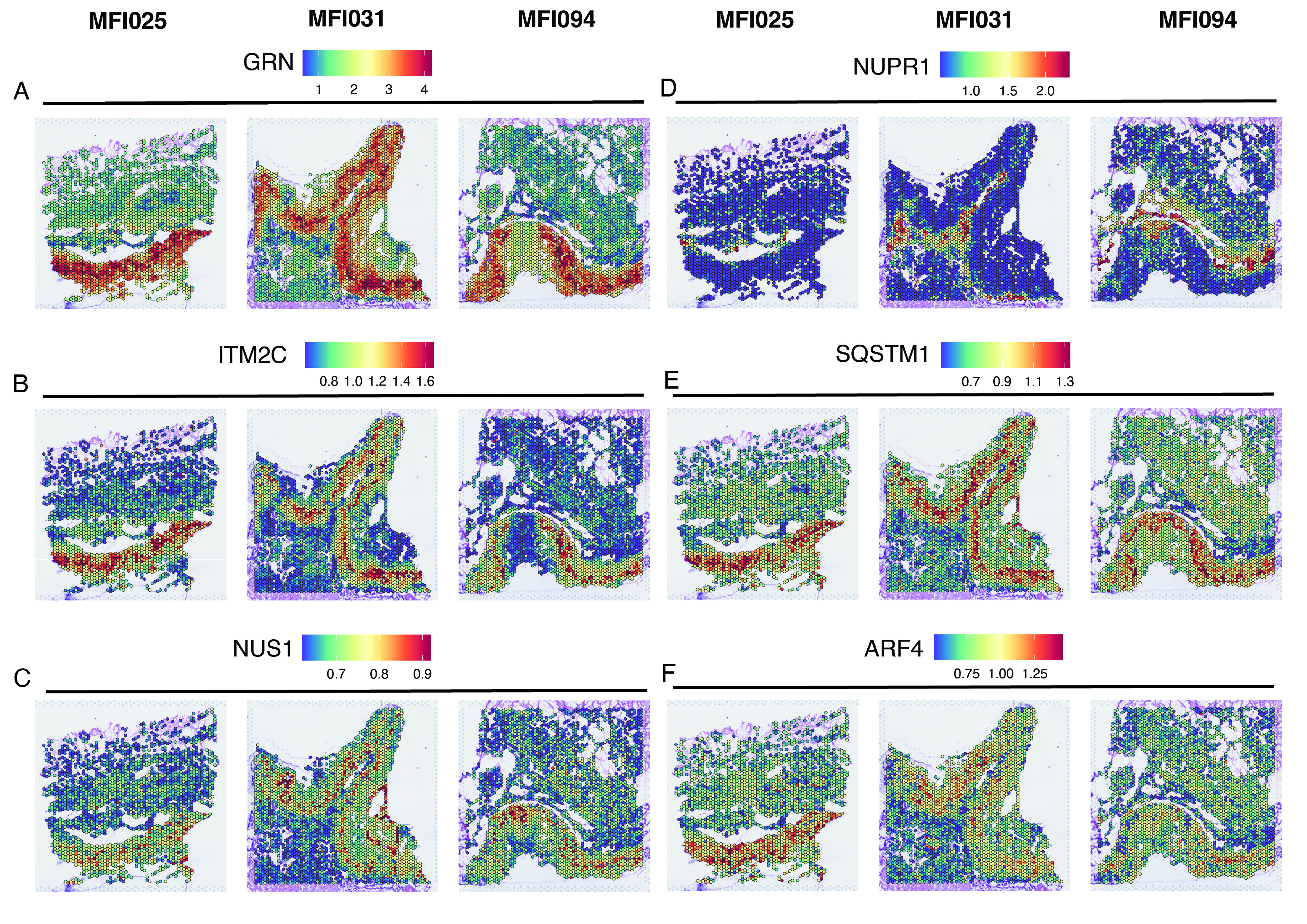

Supplement: S5 Fig — A–F) Spatial feature plots showing genes that are enriched during the transition to Areola-2 trophoblasts and their expression pattern within our spatial transcriptomics dataset, split my sample (MFI). A) GRN, B) ITM2C, C) NUS1, D) NUPR1, E) SQSTM1, and F) ARF4. (S5_Fig.JPG) [file pbio.3003302.s005.jpg]
