# Supplementary material for: Defining cellular diversity at the swine maternal–fetal interface using spatial transcriptomics and organoids
Source: PLoS Biol. 2025 Aug 28;23(8):e3003302. doi: 10.1371/journal.pbio.3003302 (PMC12393714; doi:10.1371/journal.pbio.3003302)
Supplement: Attachment — Submitted filename: Response_to_Reviewers.pdf [file pbio.3003302.s021.pdf]

*We thank the reviewers for their thoughtful comments. We have substantially revised the manuscript in response, which we believe has significantly strengthened its clarity and impact. Below, we provide point-by-point responses to each comment in blue.*

Reviewer #1:

- Figure 1H. As presented this figure does not necessarily support the conclusion that "sTOs were more similar to placental tissue than PTr2 cells…" (line 158-159). In fact, if looking at Dim 1, which counts for the highest percentage of changes among samples, PTR2 sits in between TO and placental samples. Dim 2 is the factor that separates PTR2 from the other samples. However, other dimensions are not shown that might further separate TO from placental samples in a similar manner as dim 2 for PRT2. Analysis like K-means or clustertree (or others) are required to sustain the conclusion above.

We agree that our interpretation may have been too broad in this instance. We further agree that our PCA analysis may have been unintentionally misleading. To address this, we have removed the PCA analysis from the revised manuscript and refined the language in this section to focus more specifically on our heatmap analysis as a metric of trophoblast specific gene expression rather than global analyses. Notably the text has been modified as follows:

*Lines 180-183:*
*"This analysis revealed that sTOs also express high levels of canonical swine trophoblast markers, at similar levels to porcine placental tissue., whereas PTr2 cells exhibited very low levels of the same markers (Figure 1K)."*

Further the conclusion for this section has also been altered.
*Lines 186-188:*
*"Together, these data indicate that sTO are composed of fetal-derived swine trophoblasts and display higher expression of these markers than conventional models such as PTr2 cells."*

- Figure 1I. How were these genes selected? They seem to contain a mix of trophoblast and stromal markers. A clear labelling of the cell types these markers represent in the figure would help with the interpretation of the data. Moreover, the data do not fully support the conclusion that "sTOs also expressed canonical markers of swine trophoblasts from placental tissue, whereas PTr2 cells did not (Figure 1I)". Some placental genes are equally not expressed in both PTR2 and sTO. Others are expressed in sTO but not at the same level as placental samples. Moreover, sTO seems to express genes not found in placental samples, some of which are in common to PTR2 and some are unique to sTO. A better representation of these data might also help to support the conclusion that "Together, these data indicate that sTO are composed of fetal-derived swine trophoblasts." (line 163-164). At this point this conclusion is only weakly supported by the data. Considering that the original cell suspension likely included both trophoblast and non-trophoblast cells, including fetal stromal cells and maternal decidual cells, it would be important to strengthen evidence that the derived sTO only comprise trophoblast cells.

To simplify and strengthen these findings we have made substantial alterations to this figure. First, we incorporated porcine uterine tissue bulk-rnaseq into this analysis enabling us to determine if there was uterine contamination in our sTO cultures. Furthermore, we now include known established markers of uterine glands/uterine epithelium (MUC1, MUC16, FOXA2, and WFDC2), fibroblasts (vimentin, COL1A1, CD34, and PDGFRA), and a reduced list of more established trophoblast markers (PAGE4, CTSD, CTSZ, TP63, CYP11A1, HAND1, GATA3, GATA2, and

CDH1). We additionally include hierarchical clustering analysis in this heatmap to cluster samples that have more similar expression of these markers. As shown (Figure 1K) uterine samples cluster tightly with high expression of uterine gland/epithelium markers as well as fibroblast markers. The expression of uterine gland/epithelium markers remains low in all other sample types. The placenta samples do express some level of fibroblast markers, presumably due to the presence of the interstitium in these samples. Notably, the placenta and sTO samples express moderate to high expression of these trophoblast markers, whereas PTr2 cells and uterine cells display negligible expression. Taken together, we are confident in concluding that sTOs 1.) are composed of a population of trophoblast cells that 2.) express human/swine trophoblast markers at a more similar level to placental tissue than PTr2 cells. This information coupled with our single cell RNA-seq (which confirms that sTO are a pure, highly homogenous population of trophoblasts allows us to confidently make the conclusion that sTOs are a robust, pure population of trophoblasts.

In response to this comment the following textual changes have been made in lines 166-188:

*"Having confirmed that sTO were indeed fetal derived, we then compared their expressional profile to publicly available expressional datasets of full-term porcine placental tissue, porcine uterine tissue, and PTr2 cells, a commonly used in vitro model of swine trophoblasts. This approach allowed us to compare the global transcriptional landscapes of organoids and their corresponding tissues, identifying key expressional patterns to indicate how closely sTOs mimic the native tissue profile. This analysis revealed that sTOs also express high levels of canonical swine trophoblast markers, at similar levels to porcine placental tissue., whereas PTr2 cells exhibited very low levels of the same markers (Figure 1K). Conversely, we found that while uterine tissues expressed high levels of known uterine gland and fibroblast markers, sTO displayed an absence of expression of these markers. While term placentas did exhibit some expression of fibroblast markers, they similarly expressed an absence of uterine gland markers (Figure 1K).Together, these data indicate that sTO are composed of fetal-derived swine trophoblasts and display higher expression of these markers than conventional models such as PTr2 cells."*

- Figure 2A. what is the base for choosing 5 clusters instead of a lower or higher number? The cell population is quite tight and there are no clear cluster boundaries, so the choice of cluster number seems arbitrary. The rest of the data in figure 2 also does not sustain the choice of 5 clusters. A rationale for this choice should be presented and backed up by data.

We appreciate the opportunity to clarify this point. We chose this resolution for 2 primary reasons: 1.) this resolution gave clear delineation of clusters by markers with minimal marker overlap between clusters and 2.) this resolution gave the most robust mapping to tissue in the probabilistic modeling analysis (Figure 6).

We have amended the methods section to clarify this point (lines 1405-1407):
*"This resolution was selected because it was able to clearly resolve clusters by markers, with minimal overlap in markers between clusters, and to optimize probabilistic mapping to tissue in downstream analyses."*

- Figure 2C. ELF3 and FGFR2 are not canonical trophoblast markers, and they are not highly expressed in the dataset (mean around 1-1.5). ELF5 is a trophoblast progenitor marker in human and mouse. In human, ELF3 is expressed in EVT and decidual cells. Literature suggests that FGFR2 is only expressed at low levels in normal swine maternal-fetal interface [Edwards et al 2011 Reprod Biol Endocr.]

In response to this reviewer's comments we have amended the markers in Figure 2C/D to include more traditional trophoblast markers, as well as canonical markers of uterine glands and uterine epithelial cells to rule out contamination. As shown these new markers continue to solidify our conclusion that sTO are definitively composed of trophoblasts with no stromal or glandular contamination from the maternal compartment.

- Figure 2F. Choice of trophoblast subtypes not supported by literature: SLC2A1 has low expression in swine trophoblast cells and highly expressed in maternal epithelial cells [Kramer et al. 2020]; SLC2A3 do not seem to be specific to areola cells [Kramer et al. 2020], lack of reference for ITGB3: based on Frank et al 2010 (BOR) it looks specific to areolas in mid/late-gestation.

The reviewer is correct that SLC2A1 and SLC2A3 were both chosen as markers based on Kramer et al 2020 and a review of this paper has confirmed that our selection of these markers is appropriate. For SLC2A1, Figure 8 of Kramer et al 2020 clearly demonstrates that while expression of this marker is in the uterine epithelium, it is also highly expressed in the D60 chorionic epithelium. Notably the authors comment that "*SLC2A1 protein was highly expressed in the chorionic epithelium (CE) by day 60 of pregnancy except at the tips of uterine-placental folds extending into the allantois and in areolae*". This region corresponds to the cuboidal cell populations of the porcine trophectoderm, as described in our manuscript. Given that we provided compelling evidence that sTO are not maternally contaminated (Figure 1J,1K Figure 2A,C,D,F) we feel confident that we are able to use this marker to denote this cell population. Similarly, while we agree that SLC2A3 is not specific to areola cells across all gestational timepoints, Kramer et al 2020 does demonstrate that the areola displays a marked increase in SLC2A3 expression by gestational day 60 (Figure 5; Kramer et al 2020). Given that our organoids were derived from mid-gestation placentas, we believe this is an appropriate marker choice. Similarly, we believe that this same logic allows us to utilize ITGB3 as a marker here as well. To address these comments, we have added a reference for ITGB3 to our manuscript.

- Figure 2G. Data do not fully support the conclusion that "these various sTO cell populations are distinctive lineages rather than contaminating cell populations". Data on KRT18 were already shown in Fig. 2C and expression levels of CTSB are lower from the middle part of the UMAP cloud to the bottom suggesting at least cell heterogeneity.

We respectfully disagree with the reviewer's interpretation of our data. While some degree of heterogeneity in CTSB expression is seen within our UMAP, no study has quantitatively determined the cellular heterogeneity of CTSB expression within porcine trophoblasts. While some degree of stochasticity of CTSB expression is observed within our organoids, only 3.9 percent of the 18,259 cells we sequenced had zero reads mapping to CTSB. While studies have demonstrated (via *in situ* hybridization and qPCR) that CTSB expression is high in trophoblasts *in vivo*, how these levels correlate to an *in vitro* system without supplemental hormonal signals remains unclear. Furthermore, while KRT18 was shown in Figure 2C, the localization across clusters was not shown, making this a critical figure showing that KRT18 and CTSB were not cluster specific (i.e. not localized to a minor cellular population). Lastly, the fact that known markers for various trophoblast populations were more abundant in different clusters (Figure 2F) suggests that different trophoblast populations are present within sTO, further supporting our conclusion.

- Line 292-293 "Although these markers partially resolved the clusters, their leaky expression across multiple clusters limited our ability to accurately assign cellular identities.": would a lower

number of clusters potentially help with cleaner signature? Or does this suggest that sTOs do not fully recapitulate in vivo conditions to allow specific sub-type cell formation but instead promote hybrid gene expression?

We performed this analysis at varying resolutions, both higher and lower, and found that the most robust clustering and probabilistic modeling occurs at this resolution. Notably, higher resolutions give a rare cell clusters that fail to map confidently to various structures. At lower resolutions, there are broader clusters with no improvement of probabilistic modeling based assignments. To that end we chose this resolution (0.2) because it optimized cluster segregation by markers, probabilistic modeling, and increased our ability to assign cluster identities. Due to these considerations, we believe this is an appropriate choice of resolution.

To address the second question, we have addressed the point of hybrid gene expression in other comments. We believe that hybrid gene expression naturally occurs along a stem differentiation trajectory, therefore it is still a physiologic system. Solely having terminally differentiated cell populations in an organoid system would render this model unpassage-able, which would limit its utility as an *in vitro* system. Future studies in our lab aim to identify signals whereby areolar or interface trophoblast specific differentiation can occur in this system thereby allowing for more cell type specific studies to be performed.

- Fig. 5D-E. Cluster 2 did not map to any trophoblast cluster. However, this analysis only included the placental cell of trophoblast origin. They might be non-trophoblast cells. Maybe interstitial? Do they cluster with any of the other placental cell types not included in this analysis?

Cluster 2 did not map to any placental trophoblast cluster, which the reviewer suggests raises the possibility that it might represent non-trophoblast cells. However, this is unlikely. The UMAP of single-cell data from the organoids shows a homogeneous population with strong and uniform expression of trophoblast markers, and no detectable stromal or interstitial markers. This suggests that Cluster 2 is not composed of non-trophoblast cells. Instead, Cluster 2 expresses high levels of *MKI67* and *TOP2A*, canonical markers of proliferative stem cells. Given that organoid maintenance depends on a self-renewing stem cell population, this interpretation is consistent with known biology. The reason these proliferative stem cells do not map back to the tissue dataset likely reflects their rarity *in vivo*. *MKI67*+ cells are present in the placenta but are relatively scarce. Additionally, spatial transcriptomics lacks single-cell resolution, so proliferative signals from rare cells like these may be diluted by surrounding tissue signals, preventing accurate mapping to specific cell types. Thus, while these cells exist in the tissue, current methods may not detect them with sufficient resolution to assign a clear identity.

- Fig. 2B and 5H show the same data with different colors but the proportion of cell by definition is the same so it is not introducing new information.

While the underlying data in Figures 2B and 5H are the same, we chose to re-display the plot with updated cluster names and colors in Figure 5H to improve clarity and reader comprehension in the context of the downstream analysis. Although redundant in content, this stylistic choice reinforces the narrative flow and aids interpretation for readers, particularly when transitioning between datasets. We believe this enhances readability and is appropriate for clarity.

- Both sTO clusters 1 and 3 only share partial non-overlapping gene expression signature with placental areola 2 cells. This suggests that sTOs only partially recapitulate cell types in vivo.

We agree that sTO clusters 1 and 3 share only partial, non-overlapping gene expression signatures with placental areola 2 cells. However, this observation aligns with the biology of organoid systems. As stem cell–derived models, trophoblast organoids differentiate into a spectrum of cell states. It is expected that, both *in vitro* and *in vivo*, cells along a differentiation trajectory will display hybrid expression profiles—reflecting intermediate states between progenitor and fully differentiated identities. In our system, clusters 1 and 3 likely represent cells in transitional states between proliferative stem-like trophoblasts and differentiated subtypes such as areolar or interface trophoblasts. These intermediate profiles naturally lead to partial overlaps in marker expression, consistent with the idea that differentiation is a continuum rather than a discrete event. Furthermore, it is important to recognize current limitations in single-cell data from the porcine placenta. While early gestation placentas have been profiled, areolar trophoblasts are not well-represented, and to our knowledge, no high-resolution single-cell studies have defined the full heterogeneity of interface and areolar trophoblasts *in vivo*. As such, direct one-to-one mapping is currently constrained by the absence of reference data. Our dataset contributes an important step toward resolving this gap. By characterizing organoid-derived trophoblast populations at single-cell resolution, we provide a foundational expression map that can serve as a reference for future studies. These comparisons will be strengthened as new datasets from later gestation porcine placentas become available. We anticipate that future single-cell profiling of porcine trophoblasts will enable more precise comparisons and further validate the findings presented here.

- Supplemental Figure 4C-F. GO analysis data are either too small or at too low resolution to be read. Unfortunately, I was not able to verify the results presented in the text and its conclusions.

To address this, we have removed these plots from our Figure and replaced them with supplemental tables containing all GO-terms identified in this analysis. (Supplemental Tables 7-10)

- Lines 364-368. Suppl. Fig. 4G indicates that endosomal genes are specific to areola-2 trophoblast cells. However, expression in sTOs clusters is not specific to the areola cells but expression is distributed between the areola-2 and ProlTroph with significant albeit lower expression in IntTroph cluster. This points again to the presence of hybrid cells in sTO posing a huge limitation to the use of sTO to study swine placental biology.

By nature, organoids are stem cell derived structures that differentiate into various cell types. As is with all stem cells, as differentiation occurs (both *in vivo* and *in vitro*) it is expected that cells will display varying degrees of differentiation, which would appear as "hybrid" expressional profiles. It is true that in our system we similarly have cells that fall along a differentiation trajectory falling somewhere between a proliferative stem cell and an interface-trophoblast or areolar trophoblast. It is highly likely that these cell populations exist in the porcine placenta; however, to our knowledge the single cell resolution of interface and areolar trophoblasts has yet to be performed on the porcine placenta. While early gestation placentas have been studied, areolar trophoblasts are not identified in these datasets; therefore, less is known about the areolar trophoblasts at the single cell level. Furthermore, we believe that this heterogeneity is not a weakness of our model, but rather a strength of our model. Notably, since these cells spontaneously differentiate, we can leverage this to identify what triggers differentiation to areolar trophoblasts. This could be used to increase areola formation in vivo, thereby enhancing fetal nutrient acquisition.

- The premise of figure 7 (lines 414-422) is to use CellChat to investigate maternal/fetal crosstalk

using sTO. However, the authors claim sTO are only comprised of trophoblast (fetal) cells therefore by definition it is not possible to study maternal/fetal crosstalk using only this model. However, it is possible to address that question in the Visium dataset. Therefore, figure 7 tries to combine two fundamentally different questions: (1) what are the crosstalks in the maternal/fetal interface in porcine placenta? (Visium dataset); and (2) do sTOs recapitulate the crosstalk within the trophoblast populations observed in vivo? These two questions should be treated separately in the story and data from the two datasets should not be presented in parallel.

We appreciate this feedback. To do this we have substantially altered this figure. As suggested by the reviewer we split the CellChat figure (Figure 7) into two figures. The first (Figure 5 in the revised manuscript) delves into the cell-cell interactions in the porcine maternal-fetal interface. The second (Figure 8 in the revised manuscript) segments the Visium dataset into just trophoblast populations for CellChat and compares them to the sTO communication networks. This allows for a much easier comparison of cell-cell interactions in trophoblast populations without the noise of the maternal system.

- Moreover, the Visium dataset suggests the highest number/strength of interaction occurs with maternal cells. Even when only addressing the trophoblast crosstalk in Fig. 7G-N, the presence of maternal information distracts from the trophoblast only data in the placental samples (which is the focus of the comparison with sTO). To address the second question, sub-setting the Visium dataset including only the trophoblast cells might be a better strategy to compare to sTO.

We have substantially altered this figure as discussed above.

Minor:

- The initial cell suspension is likely composed of both trophoblast and non-trophoblast cells, likely fetal stromal cells and potentially maternal cells. While some data suggest the derived organoids are trophoblast in origin, it would be important to present some characterization of the original cell suspension to compare to the cell population in established organoids.

We appreciate the reviewer's concern but respectfully disagree with the implication that characterization of the initial cell suspension is required to validate the identity of the derived organoids. Our organoid derivation strategy follows established protocols from both human and non-human placental organoid studies (e.g., Haider et al., Turco et al., Yang et al, Caldwell et al, and others), which do not require prior purification of trophoblasts or stem cells. These protocols rely on selective culture conditions to enrich for proliferative stem-like trophoblasts capable of self-renewal and organoid formation. While the initial placental digest likely includes a heterogeneous mix of fetal cells, potentially including stromal and glandular epithelium, the media formulation used strongly favors trophoblast stem cell expansion. Non-stem, differentiated cells, including stromal contaminants, typically do not survive prolonged culture or serial passaging (2–3 weeks in our system). Organoids that do form are subsequently selected by morphology and manually picked using a fine needle, allowing removal of residual fibroblasts or debris. This selection step is a standard practice in the field to ensure the propagation of epithelial organoids while excluding non-organoid structures.

Importantly, our single-cell RNA-seq data confirm that the resulting organoid cultures are composed exclusively of trophoblast cell types. We observe:

- Strong and consistent expression of canonical trophoblast markers;
- Absence of stromal (e.g., *VIM*, *COL1A1*) and glandular epithelial markers (e.g., *FOXA2*, *MUC1*, *MUC16*);
- No expression of maternal-specific markers;

- No UMAP evidence of outlier populations that would suggest contaminating lineages.

Furthermore, if glandular epithelial stem cells were present and expanding, we would expect distinct cystic morphologies in Matrigel, which we do not observe. Taken together, these findings strongly support the conclusion that although the starting material was heterogeneous, our culture conditions, passaging strategy, and selection approach effectively yielded a homogeneous trophoblast organoid population. We believe this workflow is consistent with best practices in the field and sufficiently validated by the transcriptomic data presented.

- Fig 4A. It would be useful to include the labelled maps (Fig.3D) for easily referencing the cell types in the different placentas. (also applies to Suppl. Fig 2)

We have amended the figure to include this change.

- Fig 5C is the same as 2A.

While it is true, we believe that including the renamed plot allows for enhanced readability and understanding for people reading the paper. We believe that this stylistic choice is therefore appropriate.

- Fig 5D-E. It would be useful to include the labelled maps (Fig.3D) for easily referencing the cell types in the different placentas.

We have amended the figure to include this change.

- Line 387. Supplemental Figure 7 not provided. Is this actually suppl. Fig 5?

We apologize for this typo. Indeed this should have referenced supplemental Figure 5 rather than supplemental Figure 7. We have corrected this error in the text.

- Figure 6A. text indicates that the trajectory analysis was applied after sub-setting the dataset to include only ProlTroph, IntTroph, and Areola-2 cells. However, the top part of the figure shows all the clusters, and it seems contradictory.

We thank the reviewer for this point and have amended the figure to include a UMAP with only the populations included in the analysis.

- Fig. 6B. too many genes on the side of the heatmap makes it difficult to read and find any.

We thank the reviewer for this point and have amended the figure (Figure 7) to display a reduced number of markers with a larger font size to enhance readability.

- Suppl. Figure 6. The panels are not referenced in order in the text

This figure has been removed and placed in Figure 5 and 8.

- Supplemental Fig. 6. The legend does not clarify what the labels on the side of the heatmaps represent: general signaling pathways or genes? Shouldn't incoming patterns be represented mainly by receptors?

This figure has been removed and placed in Figure 5 and 8. The legends of the heatmaps indicate the pathway and its constituents. i.e. one pathway being present often relies on multiple receptors and ligands being present. An incoming pathway would indicate that the receptor(s) for a pathway is present on the population. We have amended the figure legends for both of these to more clearly indicate this point.

- Line 452-454. This is a circular argument. The spatial data was used to predict the ligand-receptor interaction so it cannot be used also to validate the CellChat findings.

This section of the manuscript has substantially changed since the initial review and this region of the text has been removed.

- Fig. 7N. genes too squeezed and not clearly legible even when zoomed in

We have corrected this error.

- Lines 454-464/Fig 7I-J-M-N. Text should present data in order in the figure. GNR first, MK afterwards.

We have amended the figure to be consistent with figure order presentation.

- Line 605. "within" repeated

We have corrected this error.

Reviewer #2:

While the study is important, the data is not available for review and Github code is empty. As a standard practice, the author needs to make the data and code available for review. In addition, the manuscript lacks sufficient detail regarding data analysis pipelines, making it challenging to evaluate the rigor of the findings. Importantly, the authors did not reference a closely related paper "Cross-Species Insights into Trophoblast Invasion During Placentation Governed by Immune-Featured Trophoblast Cells," which provides valuable insights into early pig placentation at the single-cell level. Including this reference and contextualizing the findings would strengthen the manuscript's contributions.

I recommend a revision for this manuscript and I would be delighted to read the revised manuscript. Data and code availability. It is unusual to submit a single-cell atlas manuscript while the data and codes are not available to review, which is not the standard practice. Please check with the manuscript submission policy of the journal.

We thank the reviewer for their comments and support of the manuscript revision. We agree that the availability of data and code is essential for transparency and reproducibility, particularly in single-cell atlas studies. We have now made all raw and processed data, as well as the analysis code, publicly available in accordance with the journal's data availability policy. Accession numbers and repository links are provided in the revised Data and Code Availability section.

We also appreciate the reviewer's suggestion regarding the relevant reference. This article was intended for inclusion but was mistakenly omitted. We have now cited it in both the Introduction and Discussion and revised the text to better contextualize our findings in light of this prior work.

Lines 97-101:
"Lastly, recent studies in early gestation porcine placentas has revealed subpopulations of trophoblasts, which display unique expressional and putative functions [25,26]. While these data serve as a critical advancement, these studies are unable to spatially resolve these populations to known structures such as the interface or the areola, thereby limiting the interpretation of these data"

Lines 1202-1207:
"Previous studies have used single cell RNA-sequencing to characterize the porcine uterus and embryos[25,26,45,57]. However, these studies rely on existing markers or GO-term based functions to assign cellular identity, lack histological structural context, do not provide spatial segregation of gene expression signatures, and were not performed at a gestational stage where critical structures, such as the areola, are present. Our data robustly define the expression profiles of various histological structures at the swine maternal-fetal interface."

Additionally, in figure 2F we have included additional markers from this paper to highlight that sTO possess diverse cell populations.

Bulk RNA-seq Analysis Lines 782-784: It is unclear which analyses were performed using DESeq2. As detailed in the DESeq2 manual… DESeq2 expects raw, un-normalized counts as input, not RPKMs. The authors should clarify their input data and ensure that the pipeline aligns with best practices.

We apologize for the unclear methods. For all analyses (including DESeq2) the appropriate inputs were utilized. We have amended the methods to clarify this point and have disclosed all scripts via GitHub for review.

Lines 1344-1337:
"Raw reads were aligned to the swine genome (susScr11) using QIAGEN CLC Genomics (v20) and mapped data were exported and compiled. The packages tidyverse, magrittr, pheatmap, rio, factoextra, ggforce, tidyheatmap, and DESeq2 were utilized for analysis. All analyses were performed using package specified methods."

scRNA-seq analysis
Line 850-851. The manuscript does not specify the adjusted p-value threshold used. Including this information is critical for evaluating the robustness of differential expression analyses.

We appreciate the opportunity to clarify our methods. In lines 1411-1412 we included the following alteration to our text:

"*For marker analyses the default Seurat adjusted p-value of $p < 0.01$ was considered significant*."

We similarly included the same modification in lines 1485-1486 for the spatial transcriptomics text.

Differential Expression and GO-Term Analysis Line 868. The authors used a p-value < 0.01 threshold for differential expression. However, when performing multiple hypothesis testing, it is

recommended to use adjusted p-values to control for false discovery rates. Please justify this choice or revise the analysis using corrected p-values.

We apologize for the confusion. We used an adjusted p-value for this analysis, which is true for all analyses in this paper. Notably, these analyses utilized a Bonferroni correction to account for multiple hypothesis testing.

We have included the following alterations to the text to clarify this point lines (1428-1429): "Significant hits were defined as having a Log2FoldChange of at least 0.25 and an adjusted p-value < 0.01 (Bonferroni correction)."

CellChat Analysis for scRNA-seq Line 874-888. It is unclear how CellChat was applied to pig data, as the software defaults to human and mouse gene sets. Did the authors identify human-pig one-to-one orthologs and use only this subset of genes for the analysis? Detailed methods should be provided, as the GitHub links mentioned in the manuscript are empty or not informative to replicate the analysis. Visium analysis

We thank the reviewer for this comment. Previous groups using CellChat on porcine tissue have utilized human based annotations for the prediction of cell-cell communications (https://doi.org/10.3389/fcell.2022.796358). We therefore used a similar strategy for our analysis. We recognize that if these annotations are incomplete, these interactions would not be detected in our system. Therefore, our analysis represents a conservative estimate of cell-cell communication networks. Additionally, these findings represent a hypothesis generating step, which we believe can serve as a basis for follow up studies aiming to validate these interactions. We have amended our discussion to include a brief discussion of this point (see below). Our previous links had privacy restrictions that we have now updated, so all data/scripts should be available. We apologize for the confusion.

Lines 1096-1131:
*"Importantly, these analyses were based on known human receptor-ligand interactions and rely on shared annotations between the human and porcine genome. It remains possible that some receptor-ligand pairs in the porcine genome are not annotated, which would preclude them from being included as a interaction in our results. Therefore, this represents a conservative estimate of cell-cell communication in the porcine placenta. We believe that these data provide a robust framework for hypothesis generation for understanding signaling within the porcine placenta."*

Lines 912-915. The decision to merge, integrate, and then re-merge Visium datasets appears methodologically incorrect. Integration already accounts for batch effects. The authors should justify this approach.

We appreciate the reviewer's attention to this point and apologize for any confusion caused by the original description of our workflow. To clarify: we did not perform Seurat's IntegrateData() function, which is typically used for single-cell datasets to correct for batch effects across diverse samples. To our knowledge, integration using this method is not standard practice for Visium spatial transcriptomics data. Instead, our workflow involved merging individual Visium datasets, followed by splitting the merged object to allow SCTransform normalization and regression of technical covariates (nFeature_RNA, nCount_RNA, and percent.rb) on a per-sample basis using lapply(). We then used SelectIntegrationFeatures() solely to identify shared variable features across samples prior to re-merging with merge(). This approach allowed us to perform dimensionality reduction and clustering based on consistently variable genes, not integrated data.

To assess the robustness of our results, we also re-analyzed the data using a standard Seurat workflow:

- Merging all samples;
- Splitting for per-sample SCTransform normalization with the same regressors;
- Remerging without SelectIntegrationFeatures();
- Identifying variable features post-merge and proceeding to PCA and clustering.

Notably, this alternative approach yielded highly concordant results, including similar cluster structure, marker gene identification, and downstream probabilistic modeling outcomes. We interpret this consistency as evidence of the robustness of our findings across analysis strategies. To avoid further confusion, we have revised the relevant methods section in the manuscript (Lines 1474–1480) for clarity and have made the full analysis code available on GitHub for transparency and reproducibility.
Revised Methods (Lines 1474–1480): "*Filtered feature-barcode matrices were imported and filtered to retain cells with more than 200 detected genes and fewer than 20% ribosomal content. Individual samples were merged, split, and normalized using SCTransform() with regression of percent.rb, nFeature_RNA, and nCount_RNA. Shared variable features were identified using SelectIntegrationFeatures() to guide consistent dimensionality reduction. Datasets were re-merged using merge(), followed by dimensional reduction and clustering via RunPCA on the top 50 PCs".*

Before integration, did the authors run PrepSCTIntegration? This is a required step for SCT-based integration.

We apologize for the confusion. We did not perform integration on the Visium spatial transcriptomics data. Rather we did this for the sTO dataset, which did utilize a PrepSCTIntegration step prior to integration.  We have amended to text to better reflect the analysis and have released the script via github.

Lines 1474-1480:
"*Individual imported filtered feature matrices underwent filtering for to only include cells with nFeatures falling above 200 and percent ribosomal reads lower than 20. Matrices were then merged, and individually normalized using SCTransform, regressing percent ribosomal reads, nFeatures, and nCounts. Variable features across the datasets were then obtained using SelectIntegrationFeatures and datasets were remerged using Merge_Seurat_List. Variable features were then set using VariableFeatures and dimensional reduction and cluster analysis performed using RunPCA on the top 50 dimensions*".

Line 914. Which method was used for the integration? SelectIntegrationFeatures alone does not complete the integration process.

We apologize for the confusion. We have amended to text to better reflect the analysis and have released the script via github.

Lines 1474-1480: "Individual imported filtered feature matrices underwent filtering for to only include cells with nFeatures falling above 200 and percent ribosomal reads lower than 20. Matrices were then merged, and individually normalized using SCTransform, regressing percent ribosomal reads, nFeatures, and nCounts. Variable features across the datasets were then obtained using SelectIntegrationFeatures and datasets were remerged using Merge_Seurat_List.

Variable features were then set using VariableFeatures and dimensional reduction and cluster analysis performed using RunPCA on the top 50 dimensions".

Line 915. After integration, did the authors set the default assay to "integrated"? Additionally, why were new variable features obtained post-integration? This requires clarification.

We apologize for the confusion. In this analysis we did not actually integrate these datasets, rather we simply merged them. We have amended to text to better reflect the analysis and have released the script via GitHub.

Lines: 1477-1480

*"Variable features across the datasets were then obtained using SelectIntegrationFeatures and datasets were remerged using Merge_Seurat_List. Variable features were then set using VariableFeatures and dimensional reduction and cluster analysis performed using RunPCA on the top 50 dimensions."*

Line 922. The manuscript does not specify the adjusted p-value threshold used. This information is essential for evaluating the rigor of the analysis.

We appreciate the opportunity to clarify our methods. In lines we included the following alteration to our text:

Lines: 1485-1487

"For marker analyses the default Seurat adjusted p-value of $p < 0.01$ was considered significant."

Lines 932-935. Identifying markers for the integrated assay of spatial transcriptomics (sTO) is methodologically incorrect. For differential expression analysis, the assay must be set to "RNA." Please also clarify the adjusted p-value threshold used in this analysis.

We appreciate this important clarification. We have modified our analysis to identify markers from the SCT assay instead of the integrated assay. We chose to use SCT rather than the RNA assay because the label transfer algorithm used for our probabilistic modeling utilizes data from the SCT assay for predictions rather than the RNA based assay. In these marker analyses (as well as in all other marker analyses in this paper) we utilized a corrected p-value cutoff of 0.01 to demarcate significance. We have updated the figures to reflect these changes and have made the following alterations to the text.

Lines 489-570:
*"We then identified shared markers between sTO clusters and their corresponding spatial transcriptomics clusters to determine the gene expression signatures that defined these mappings. We found that sTO cluster 0 cells expressed 59 markers that were conserved in interface-trophoblasts, which included AKR1B1, HAVCR1, C1QTNF4, and TGM2 (Figure 5F, 5G, Supplemental Table 3). Similarly, sTO cluster 1 expressed 63 shared markers with areola-2 cluster, including cathepsins A and D (CTSA and CTSD), SLC7A4 and NUPR1 (Figure 5F, 5G, Supplemental Table 4). sTO cluster 3 had 110 shared markers with areola-2 cluster, with both clusters highly expressing ISG15, FTL, MDK, and B2M (Figure 5F, 5G, Supplemental Table 5).*

*Conversely, sTO cluster 4 expressed only 20 shared markers to areola-1 cluster, largely including COX1, COX3, ND4, and CYTB (Figure 5F, 5G, Supplemental Table 6). Using this approach, we were able to assign cellular identities and identify shared gene expression profiles for all trophoblast populations within sTOs, which were distributed at similar ratios across independent lines (Figure 5H). Taken together these data indicate that sTO are able to spontaneously differentiate into various populations of trophoblasts found at the porcine maternal-fetal interface."*

Lines 1496-1506:
*"To identify shared markers between sTO and their corresponding tissue structures, marker lists for the SCT assay of sTO and the SCT assay of the visium data were generated using FindAllMarkers with a minimum fold change threshold of 0.25 and detected in a minimum of 0.25 of cells, with adjusted p-values of 0.01 considered significant."*

CellChat Analysis for Visium Line 938-952. Similar to the scRNA-seq analysis, it is unclear how CellChat was applied to pig data. If the authors used human-pig orthologs, they should provide details about the identification process and the subset of genes used. As previously noted, the reported GitHub links are empty or not sufficiently detailed to replicate the analysis. Ensuring reproducibility is crucial for validating the results.

We thank the reviewer for this comment. Previous studies using CellChat on porcine tissue have utilized human based annotations for the prediction of cell-cell communications (Tian, Q. 2022 Front Cell Dev Bio). We therefore used a similar strategy for our analysis. Our previous links had privacy restrictions that we have now updated, so all data/scripts should be available. We apologize for the confusion.

Reviewer #3: Introduction- Please cite Arutyunyan et al Nature 2023 particularly in the first sentence of line 79. Also in line 87, Arutyunyan and Shannon et al Dev Cell 2024. Line 110, cite Haider et al 2018.

We have amended the text to include these citations.

Figure 1- A-D) How did you assess that you were able to isolate purely trophoblast stem cells? Please include FACS markers, IFs, that trophoblast markers are present. I realize not all human antibodies will work with pig, but please try! I don't think it's enough to say, they are proliferating as spherical organoids, therefore they are trophoblast stem cells. Especially considering your bulk seq in park I-

We appreciate the reviewer's emphasis on rigorously confirming trophoblast stem cell identity and agree that additional characterization is important. Our organoid derivation strategy was based on established protocols from multiple species, including pig, human, and cow (Yang et al., 2022; Post et al., 2020; Turco et al., 2018; Liu et al., 2024), which do not require isolation of pure trophoblast stem cells at the outset. These protocols rely instead on selective culture conditions that promote expansion of proliferative trophoblast stem-like cells while differentiated or non-epithelial cells fail to expand or are lost during passaging. In our system, organoids were passaged over 2–3 weeks and selected based on morphology using needle-picking. This step allowed us to exclude non-epithelial structures such as fibroblast aggregates. The resulting cultures were highly homogeneous and composed of proliferative spherical structures consistent with trophoblast organoids.

Importantly, we validated trophoblast identity using single-nucleus RNA sequencing, which demonstrated uniform expression of canonical trophoblast markers across all organoid clusters, including *GATA3*, *TFAP2A*, *KRT18*, *KRT7*, *CTSL*, and *CTSB*, and no expression of stromal or glandular epithelial markers (e.g., *VIM*, *COL1A1*, *FOXA2*). In response to the reviewer's suggestion, we also performed immunofluorescence staining for conserved trophoblast markers, including GATA3, KRT18, KRT7, CTSL, and CTSB, in our pig trophoblast organoids. Despite challenges with cross-reactivity of human antibodies, we were able to detect consistent protein expression for several of these markers, including robust nuclear GATA3 and cytoplasmic KRT18 and CTSB staining. These findings further support the trophoblast identity of our cultures. Taken together, our data from morphology, transcriptomic profiling, and targeted protein staining support the conclusion that the derived organoids are composed of proliferative trophoblast cells.

I) Many of the transcriptional profiles appear opposite of term placenta, and about half of it is definitely opposite of PTR2 (which is the point, I realize, but what trophoblast type does PTR2 represent?). The only regions that look like TOs match term placenta is MMP24-SOX2, WFDC2-PLET1. Could the canonical genes of interest that match placental tissue be highlighted?

We have made substantial alterations to this figure panel to simplify the interpretation. First, we incorporated porcine uterine tissue bulk-RNAseq into this analysis enabling us to determine if there was uterine contamination in our sTO. Furthermore, we only included known established markers of uterine glands/uterine epithelium (*MUC1*, *MUC16*, *FOXA2*, and *WFDC2*), fibroblasts (*VIM*, *COL1A1*, *CD34*, and *PDGFRA*), and a reduced list of more established trophoblast markers (*PAGE4*, *CTSD*, *CTSZ*, *TP63*, *CYP11A1*, *HAND1*, *GATA3*, *GATA2*, and *CDH1*). We additionally included hierarchical clustering analysis in this heatmap to cluster samples that have more similar expression of these markers. As shown (Figure 1K) uterine samples cluster tightly with high expression of uterine gland/epithelium markers as well as fibroblast markers. The expression of uterine gland/epithelium markers remains low in all other sample types. We can see that the placenta samples do express some level of fibroblast markers, presumably due to the presence of the interstitium in these samples. Notably, we see that the placenta and sTO express moderate to high expression of these trophoblast markers, whereas PTr2 cells and uterine cells display negligible expression of these markers.

H) could a maternal epithelial cell type be included in this analysis to demonstrate distinction from this cell type?

We appreciate this feedback and have included uterus bulk RNAseq from gestational D14 uterus (Figure 1K). As shown the uterus expressional profile is highly distinct from the placenta and organoids samples, highlighting the shared expressional profile of sTO and placentas.

G) The organoids are grown in matrigel droplets which do not replicate chorionic tissue architecture, and then grown in suspension, this does appear better in terms of the function of the trophoblasts and the cellular orientations, why wasn't this chosen for scRNAseq? This question is related to figure 2-
Can columnar morphology be observed in any of the organoids that indicates not just transcriptional alignment with areola, but in cell morphology as well?

We appreciate the reviewer's interest in the rationale behind our culture strategy and its relevance to tissue architecture. While suspension culture may more closely replicate aspects of chorionic villi in humans—where villi are directly exposed to maternal blood and shear forces—this is not the case in swine. In the porcine placenta, trophoblasts remain in direct contact with the maternal

epithelium, without exposure to maternal blood or fluid. Therefore, we believe that Matrigel-embedded culture more closely recapitulates the *in vivo* microenvironment of pig trophoblasts, particularly in terms of matrix contact and spatial organization. In addition, Matrigel droplet culture is the standard approach for propagating trophoblast organoids across a range of species, including human, rhesus macaque, cow, and even reptiles (e.g., Yang et al., 2022; Post et al., 2020, Caldwell et al, 2025). In our experience, suspension cultures, particularly in non-human species, are more challenging to maintain and propagate, making them less suitable for high-throughput applications such as single-nucleus RNA-seq. Importantly, the goal of our single-cell analysis was to establish a baseline cellular composition of swine trophoblast organoids (sTOs) under standard propagation conditions. In prior studies with human TOs, we have shown that organoids grown in suspension versus Matrigel droplets maintain similar cellular compositions, with only modest differences in gene expression (Keenen et al, 2025). Thus, we expect that the transcriptional identity of cells in sTOs would not be substantially altered by culture format. Regarding the second part of the question: While we have not yet performed a detailed histological or ultrastructural analysis of organoid morphology to identify columnar epithelial organization**,** the strong expression of columnar epithelial markers in specific clusters (e.g., *EPCAM*, *KRT18*, *CLDN4*) is consistent with this morphology**.** We agree this is an important future direction and are currently working to establish immunostaining protocols to more directly assess architectural features, including apicobasal polarity and columnar structure.

Figure 2- Why were the matrigel-embedded sTOs used for scRNAseq? This is never justified based on the observations of the previous figure. To me, it seems that the previous figure (1) shows that the suspension organoids replicate more relevant in vivo orientation? So, why were matrigel-embedded TOs used?

We thank the reviewer for this thoughtful question and the opportunity to clarify our rationale. While suspension culture may more closely mimic aspects of chorionic villus exposure in species like humans, where villi are directly bathed in maternal blood, this is not representative of the swine placental architecture. In pigs, trophoblasts maintain direct contact with the maternal epithelium throughout gestation, without exposure to maternal blood or fluid. As such, we believe that Matrigel-embedded culture more accurately recapitulates the *in vivo* environment for porcine trophoblasts, providing a supportive matrix consistent with their natural interface. Moreover, Matrigel-based culture is the established standard for propagating trophoblast organoids across multiple species including human, rhesus macaque, bovine, and bat species (e.g., Yang et al., 2022; Post et al., 2020, Caldwell et al, 2025, etc) due to its compatibility with long-term passaging and consistent support of trophoblast stem cell maintenance. In our experience, suspension cultures in non-human systems are more difficult to maintain and passage reliably, particularly when scaling up for downstream applications like single-cell RNA sequencing. The primary objective of the single-cell analysis presented in Figure 2 was to establish a baseline cellular composition of sTOs under standard propagation conditions. In previous studies using human TOs, we observed that suspension versus Matrigel culture yielded similar cellular compositions, with only modest transcriptional differences (Keenen et al, 2025). Based on these findings, we anticipated that the culture format would not substantially alter the core cellular architecture of the organoids. Therefore, we selected Matrigel-grown organoids for scRNA-seq as the most practical, robust, and physiologically appropriate system for pigs.

C) What about other traditional mouse/human trophoblast markers like GATA2/3 and TEAD4?
C/D) There is no stromal contamination based on this data, but what about maternal epithelial contamination? Please include markers of endoderm (assuming this is the lineage of the swine endometrium) to compare as well.

In response to this reviewer's comment (as well as other reviewer comments) we have amended the markers in Figure 2C/D to include more traditional trophoblast markers, as well as canonical markers of uterine glands and uterine epithelial cells to rule out contamination. As shown these new markers continue to solidify our conclusion that sTO are definitively composed of trophoblasts with no stromal or glandular contamination from the maternal compartment.

Figure 6- I have reservations that the differentiation progression is indeed a linear ProfTroph -> IntTroph -> Areola-2. Unless this is a well known progression that I am unaware of, your data could also indicate a bifurcating differentiation pattern from ProfTroph to IntTroph and ProfTroph to Areola-2 separately. I'm unfamiliar with this Trajectory inference analysis, but is a bifurcating pattern a possible output? If it is only programmed to recognize linear progressions, then the software will find a way to fit a lineage progression. I would like to see either a change in language in the text to the possibility that there could be a bifurcating pattern, but there is not software to recognize this, or the authors may have to perform a timecourse of multiple samples to discover whether the two lineages arise temporally simultaneously (perhaps indicating a bifurcation) or sequentially (indicating a linear progression). To me, your data as presented indicates a bifurcation,
and this also makes more sense when in previous figures, the authors describe very different functions of each lineage. Like other figures, I would like to see the authors spatially locate the transcripts within the organoids. This could be done in a timecourse, and does not need to be done with expensive multiple scRNAseq samples. Using your markers, identify with qPCR or HCR-RNAfish (or something similar) where and when these transcripts arise. Or, bulk seq multiple time points?

We appreciate the reviewer's critique and the opportunity to clarify our trajectory inference analysis. In our pseudotime analysis, we used Slingshot, which does indeed allow for nonlinear and bifurcating trajectories not only linear ones. As part of the setup, we manually defined the root at the proliferative trophoblast (ProfTroph) cluster, based on high expression of canonical stemness markers (*MKI67*, *TOP2A*), and allowed the software to infer downstream differentiation paths without further constraint. Slingshot is not restricted to fitting linear trajectories; rather, it fits principal curves that reflect the structure of the data and is capable of detecting multiple branches where supported by the underlying dimensionality. In our case, no bifurcation was detected and the best-supported trajectory proceeded from ProfTroph through IntTroph to Areola-2. However, we agree that the biology of trophoblast differentiation *in vivo* is likely more complex and may involve multiple parallel or branching pathways. While our model does not currently support a bifurcation, we acknowledge that a bifurcating progression remains a plausible alternative, particularly given the distinct functions of the interface and areolar trophoblast populations described in earlier figures.

As the reviewer notes, a definitive conclusion would require temporal sampling across differentiation or spatial transcriptomic validation within organoids. We agree these are important next steps. Although we have not yet performed such time-course studies, existing developmental data in pigs supports the notion of a sequential progression: columnar trophoblasts are observed earlier during the elongation phase, while areolar structures are typically evident later (around gestational day 24), suggesting that interface trophoblasts may form first, followed by progressive differentiation into areolar trophoblasts. This temporal separation supports a linear model, but does not rule out parallel or partially overlapping differentiation pathways.

To acknowledge this uncertainty and incorporate the reviewer's suggestion, we have revised the discussion to highlight the possibility of bifurcation and the need for future spatial and temporal resolution:

Revised Discussion (Lines 1184–1190): "*We believe this study provides a foundational framework for exploring porcine placental development, including the establishment of a physiologically relevant organoid model and a spatially resolved map of the maternal-fetal interface. These data now enable future studies aimed at testing key hypotheses. For example, whether interface and areolar trophoblasts arise through linear or bifurcating differentiation programs remains unresolved, and could be addressed through spatial transcriptomics or time-course experiments. Additionally, targeted studies of the signaling pathways implicated in this progression may offer new insights into placental biology and opportunities for improving swine reproductive health*."

We thank the reviewer again for prompting this important clarification and for suggesting valuable directions for future work.

Questions beyond the figures- Given your vast transcriptome data, could you alter culture conditions such that areolar cells could be enriched or matured?

We appreciate this suggestion and believe it is an important future direction. We believe that co-culturing of sTO with uterine glands, or uterine fluid, may provide enhanced differentiation of areolar subsets enabling more detailed analyses of what stimulates such differentiation. Identification of compounds/signals that promote this differentiation could then allow us to add media supplements in a more controlled manner to stimulate differentiation and allow for mechanistic studies into areolar trophoblasts differentiation pathways. We are currently attempting to perform these studies.

We have included a discussion about future directions and the utility of this model in the discussion section of the paper:

Lines 1184-1190:
"*We believe this study serves as a foundational study for studying the porcine placenta, via the presentation of a physiologic model and a spatially resolved roadmap of the maternal-fetal interface. These critical findings allow for future studies to be proposed. For example, future studies aimed at studying the function of the signaling pathways identified here may discover new avenues to improve swine husbandry. Studies aimed at identifying the triggers of areola development may now be possible in vitro. We hope that these data will spark new interest in studying porcine reproductive biology*."

Can you spatially resolve where the trophoblast populations reside within the organoids? How does it change when the organoids are cultured in suspension?

At this time, we have not performed spatial mapping of individual trophoblast subtypes within organoids under either Matrigel-embedded or suspension culture conditions. However, we fully agree that such analysis would provide valuable insights into how *in vitro* organization reflects *in vivo* architecture.

Spatial transcriptomics techniques, such as Visium or Slide-seq, are not readily applicable to 3D organoids due to several technical limitations. First, organoids must be sectioned and flattened

onto slides, often leading to distortion or incomplete representation of their three-dimensional structure. In addition, organoids vary in size, shape, and orientation, complicating efforts to standardize spatial readouts across samples or conditions. Second, although new platforms such as Visium HD offer greatly improved resolution that may be theoretically sufficient to resolve structures/cell types within organoids, their application is currently limited by species compatibility, notably, the lack of porcine-validated probe sets for use with commercially available spatial kits. Moreover, these platforms are optimized for flat, tissue-like structures, and applying them to 3D organoids remains technically challenging. Organoids must be embedded, sectioned, and flattened onto slides, which can result in morphological distortion or sampling bias. In addition, organoids vary in size, shape, and orientation, complicating efforts to standardize spatial readouts across samples or conditions.

To address these limitations, we are currently pursuing alternative spatial approaches, including HCR-RNA FISH and multiplexed immunofluorescence, to visualize the spatial distribution of trophoblast subtypes using conserved markers in intact organoids. These methods are more flexible and species-agnostic, and we believe they will provide complementary spatial information at single-cell resolution.
